# Convolutional neural network-based magnetic resonance image differentiation of filum terminale ependymomas from schwannomas

**DOI:** 10.1186/s12885-024-12023-0

**Published:** 2024-03-19

**Authors:** Zhaowen Gu, Wenli Dai, Jiarui Chen, Qixuan Jiang, Weiwei Lin, Qiangwei Wang, Jingyin Chen, Chi Gu, Jia Li, Guangyu Ying, Yongjian Zhu

**Affiliations:** 1 Department of Neurosurgery, The Second Affiliated Hospital, School of Medicine, Zhejiang University, 88, Jiefang Road, Hangzhou, China; 2 Zhejiang University School of Mathematical Sciences, Hangzhou, Zhejiang China; 3 Clinical Research Center for Neurological Diseases of Zhejiang Province, Hangzhou, China; 4 Ningbo Medical Center Lihuili Hospital, Department of Neurosurgery, Ningbo University, 1111, Jiangnan Road, Ningbo, Zhejiang China

**Keywords:** Convolutional neural networks, Filum terminale ependymoma, Schwannoma, Contrast-enhanced magnetic resonance imaging

## Abstract

**Purpose:**

Preoperative diagnosis of filum terminale ependymomas (FTEs) versus schwannomas is difficult but essential for surgical planning and prognostic assessment. With the advancement of deep-learning approaches based on convolutional neural networks (CNNs), the aim of this study was to determine whether CNN-based interpretation of magnetic resonance (MR) images of these two tumours could be achieved.

**Methods:**

Contrast-enhanced MRI data from 50 patients with primary FTE and 50 schwannomas in the lumbosacral spinal canal were retrospectively collected and used as training and internal validation datasets. The diagnostic accuracy of MRI was determined by consistency with postoperative histopathological examination. T1-weighted (T1-WI), T2-weighted (T2-WI) and contrast-enhanced T1-weighted (CE-T1) MR images of the sagittal plane containing the tumour mass were selected for analysis. For each sequence, patient MRI data were randomly allocated to 5 groups that further underwent fivefold cross-validation to evaluate the diagnostic efficacy of the CNN models. An additional 34 pairs of cases were used as an external test dataset to validate the CNN classifiers.

**Results:**

After comparing multiple backbone CNN models, we developed a diagnostic system using Inception-v3. In the external test dataset, the per-examination combined sensitivities were 0.78 (0.71–0.84, 95% CI) based on T1-weighted images, 0.79 (0.72–0.84, 95% CI) for T2-weighted images, 0.88 (0.83–0.92, 95% CI) for CE-T1 images, and 0.88 (0.83–0.92, 95% CI) for all weighted images. The combined specificities were 0.72 based on T1-WI (0.66–0.78, 95% CI), 0.84 (0.78–0.89, 95% CI) based on T2-WI, 0.74 (0.67–0.80, 95% CI) for CE-T1, and 0.81 (0.76–0.86, 95% CI) for all weighted images. After all three MRI modalities were merged, the receiver operating characteristic (ROC) curve was calculated, and the area under the curve (AUC) was 0.93, with an accuracy of 0.87.

**Conclusions:**

CNN based MRI analysis has the potential to accurately differentiate ependymomas from schwannomas in the lumbar segment.

**Supplementary Information:**

The online version contains supplementary material available at 10.1186/s12885-024-12023-0.

## Introduction

Filum terminale ependymomas (FTEs) are common primary spinal cord tumours of the lumbosacral segment, and their incidence is secondary only to that of schwannomas [1, 2]. Spinal magnetic resonance (MR) is the state-of-the-art method for diagnosing spinal tumours. Preoperative distinction of these two kinds of tumours via MR images is often difficult and frequently inaccurate.

**Fig. 1:**
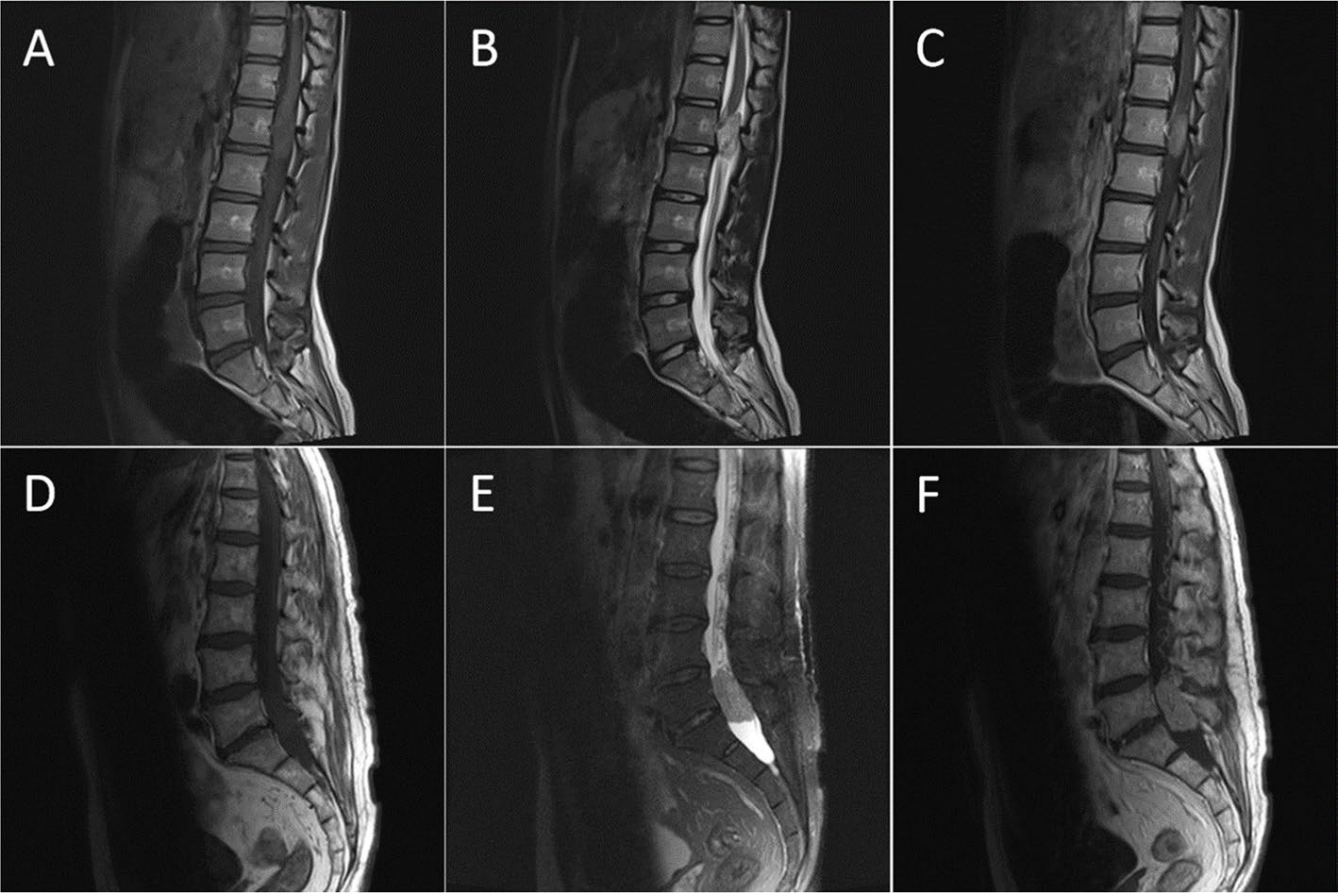
Enhanced MR images of ependymoma and schwannoma. Images **A**-**C** belong to a 13-year-old female patient who was admitted to the hospital due to left lower limb pain. Postoperative pathology indicated a spinal ependymoma, WHO Grade II. Images **D**-**F** belong to a 55-year-old male patient who was admitted to the hospital due to lower back pain and left lower limb pain. Postoperative pathology indicated a schwannoma. **A** & **D** T1-weighted image (T1-WI). **B** & **E** T2-weighted image (T2-WI). **C** & **F** Contrast-enhanced T1-weighted image (CE-T1)

According to existing radiological analyses of intradural tumours, both FTEs and schwannomas can appear as T1 iso-/hypointense and T2 hyperintense on MRI with intense enhancement [3]. Solid nodular tumours can exhibit a high degree of similarity as shown in Fig. 1. These tumours also exhibit more typical imaging features, such as long-segment intramedullary FTEs and dumbbell-shaped schwannomas. These notable features are beyond the scope of our discussion.

With the development and popularity of minimally invasive neurosurgery, most intraspinal tumours can be removed through microforaminotomy [4] after piecemeal resection (removing the tumour in small pieces), which may increase the risk of tumour metastasis in some tumours, such as ependymomas. Ependymomas are prone to spread in the spinal canal during surgery [5–7], and the total laminectomy approach is preferred. En bloc resection of the tumour and its surrounding tissue as a single piece is more ideal and leads to an improved prognosis [8, 9]. A 20-year study [10] suggested that en bloc gross total resection (GTR) should be the goal during surgery for cauda equina ependymomas. This approach can reduce recurrence, but there is no significant association between histological subtypes. However, schwannomas are more benign. A study involving 2542 adults reported that the recurrence rate of schwannomas was 5.3%, and schwannoma recurrence was associated with subtotal resection [11]. In most cases, intracapsular tumour decompression is necessary, and patients subjected to this approach have a decreased risk of recurrence after GTR [12]. Therefore, surgical approaches such as hemilaminectomy or semi-hemilaminectomy can be employed to remove tumours while minimizing the window size. The advantages of this approach include preservation of bony structures, reduced surgical trauma, and rapid postoperative recovery. Anticipation of the need for en bloc resection is crucial in preoperative surgical planning [13, 14].

In the past decade, radiomics [15], machine learning (ML) [16] and deep learning (DL) [17–19] have been proposed as effective approaches for feature extraction and classification of radiologic images [20]. DL models automatically learn feature representations, reducing the need for manual feature engineering. For medical imaging learning tasks involving massive amounts of data, convolutional neural networks (CNNs) have considerable advantages in improving training efficiency and preventing overfitting. Despite the important promise of DL in visual tasks, there have been few reports on the potential for differentiating spinal tumours. One study [21] employed CNNs to classify spinal tumours using MRI datasets. The model achieved an accuracy of 82%, demonstrating DL’s powerful capability in identifying complex patterns in medical images that are often imperceptible to the human eye. Since then, a few studies have been published on the segmentation and target detection of intradural lesions [22, 23].

In light of this, we propose a CNN model to differentiate between two tumours based on MR images. We compared the performances of three different CNN models and selected the most efficient model. We also compared models using four different MRI modalities. We tested this model on an additional external dataset. This model does not require manual delineation of tumour boundaries or selection of imaging features by doctors. To the best of our knowledge, there is currently no existing work on this topic, making it the only model capable of distinguishing between ependymomas and schwannomas.

## Methods

### Patient selection

The demographic data of the patients are shown in Table 1.

**Table 1:** Summary statistics of patient characteristics

Variable	Cohort, No.(%)	
	Ependymoma	Schwannoma
**Patient demographics**		
No. of individuals (train: test)	84(50:34)	84(50:34)
Age(mean ± SD)	50 ± 21	68 ± 12
Gender (Male: Female)	24:60	56:28
**No. of images**		
Cross-validation training dataset (T1-WI: T2-WI: CE-T1)	406 (117:165:124)	402 (112:167:123)
External test dataset (T1-WI: T2-WI: CE-T1)	230 (65:97:68)	210 (59:85:66)

We retrospectively collected preoperative enhanced MR images of lumbosacral FTEs and cauda equina schwannomas treated surgically at the Second Affiliated Hospital of Zhejiang University between 2013 and 2021. Considering intraspinal tumours in the lumbosacral spinal canal, there was a greater incidence of schwannomas in males than in females; otherwise, there was a greater incidence of ependymomas (*p* < 0.05). There was no significant difference in age distribution. Postoperative pathological results served as the gold standard for diagnosis. According to the 2021 CNS WHO classification [24], the FTE patients we included exhibited two pathological subtypes: classical spinal ependymoma (SPE, WHO II) and myxopapillary ependymoma (MPE, WHO II), without MYCN amplification. Patients with other pathological subtypes were excluded from our study.

Long-segment intramedullary ependymomas were excluded because they can be easily distinguished by growth pattern. Dumbbell-shaped schwannomas were also included in the exclusion criteria because it is almost impossible for ependymomas to grow in this manner.

### Datasets and imaging processing

The contrast-enhanced MR images included T1-weighted (T1-WI), T2-weighted (T2-WI) and contrast-enhanced T1-weighted (CE-T1) MRI sequences. All neuroimaging data were acquired using a 1.5T superconducting magnetic resonance scanner with a spinal phase coil. All patients underwent axial, sagittal and coronal T1-WI and T2-WI scans at various TR/TE values. After the injection of GD-DTPA (0.1 mL /kg), axial, sagittal and coronal T1-weighted images were acquired, and the same parameters were used for plain scanning.MR images were collected from nine MR machines, involving various types of magnetic resonance vendors such as GE MEDICAL SYSTEMS SIGNA EXCITE, GE MEDICAL SYSTEMS DISCOVERY MR750, SIEMENS Sonata, SIEMENS Aera and uMR790, at two branches of the Second Affiliated Hospital of Zhejiang University.

The sagittal sequences containing the layer of the tumour occupying the dura mater were selected from the enhanced MR images of each patient. The dataset was prepared with the image annotation tool Labelme [25] (https://github.com/wkentaro/labelme) and annotated using the rectangular annotation tool. The saved .json files were used as input for the training model. The annotation contains some developed spinal cord or circuitous filament fibres, as these areas around the tumour might be recognized as different features by the algorithm.

We selected one hundred patients, with a total of 406 ependymoma and 402 schwannoma images, as a relatively large training and internal validation dataset. An additional 34 pairs of patients newly diagnosed in the year 2022 were included in the external test groups. A total of 230 ependymoma and 210 schwannoma images were included in the test set (Table 1).

### Deep learning methods

**Fig. 2:**
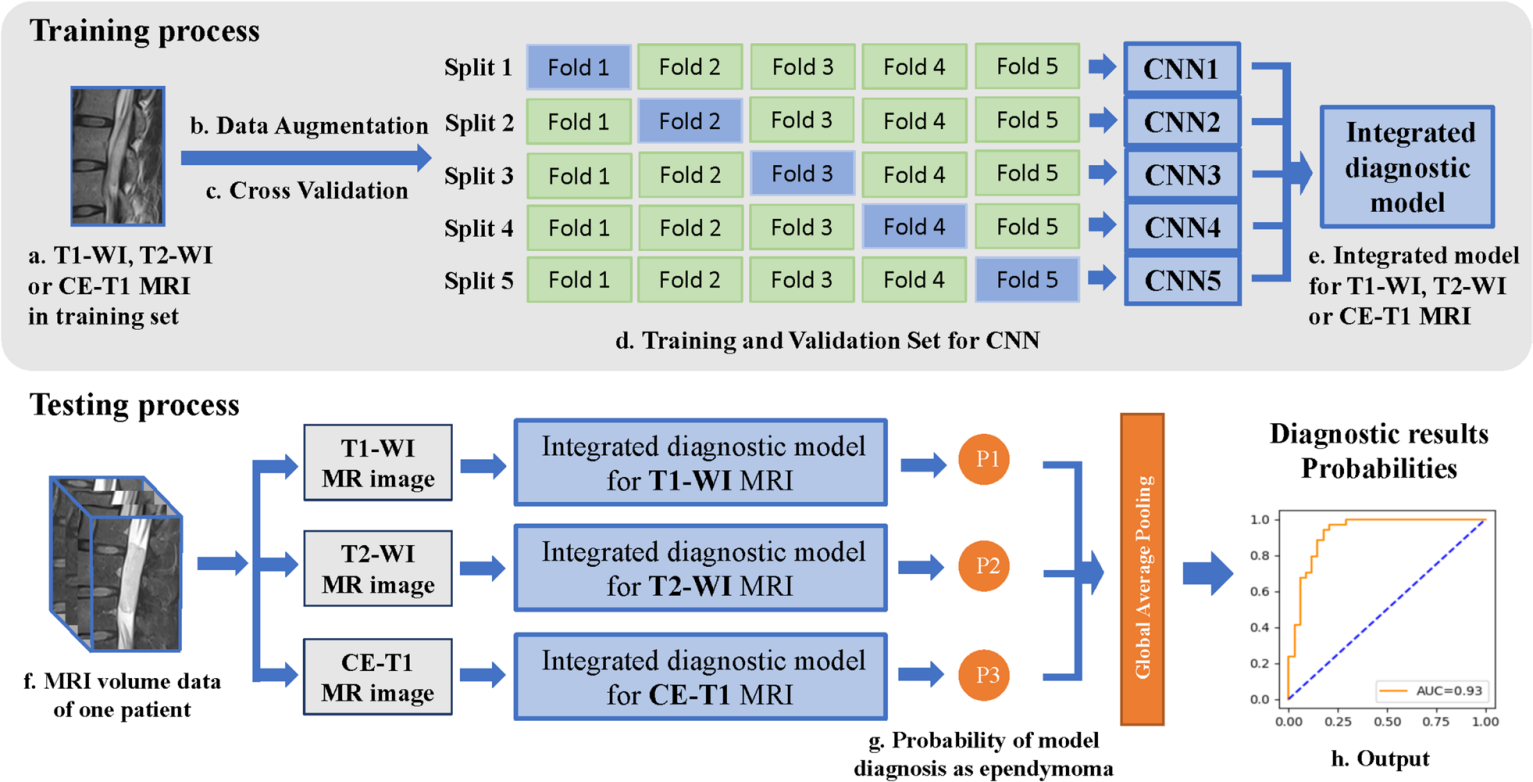
Pipeline of the proposed diagnostic system. **a** training images. **b** data augmentation consisted of random rotation, random horizontal-flip and normalization. **c** five-fold split of cross validation. **d** for every mode, five CNN models are trained at image level. **e** integrated diagnostic model from five CNN models. **f** external test images of one case. **g** probability of model diagnosis from 3 MRI modalities. **h** output of diagnostic system

The entire workflow of our tumour diagnosis system is shown in Fig. 2. In the training process, we trained specialized classifiers for different modalities, including the T1-weighted image classifier, T2-weighted image classifier and CE-T1 classifier. The dedicated classifier of a single mode was integrated by five CNN models, which were five models with the same structure obtained by conducting fivefold cross-validation on the training set as shown in Fig. 2. In other words, we evenly divide the training set of one mode into 5 parts according to the cases. For each fold of cross-validation, we selected the model with the best accuracy on the current validation set as the current fold model. Then, we obtained 5 models via fivefold cross-validation. We use average pooling on the diagnoses of 5 models to form an ensemble classifier, namely, Fig. 2e. Before training the models, we used data augmentation, which included a maximum of 40 degrees of random rotation, random horizontal-flipping, and random resizing with a scale of 1.5. Then, the grayscale images were normalized to 0.5 as the average and 0.5 as the standard deviation, and the length and width of the images were standardized to 500. After data augmentation and standardization, five diagnostic classifier models were trained for three training datasets (T1-WI, T2-WI, and CE-T1 MRI) using fivefold cross-validation (Fig. 2b). After comparing multiple backbones, including EfficientNet-b2 [26], ResNet-50 [27] and Inception-v3 [28], we ultimately chose Inception-v3 as the backbone of our model.

Consider $X={\{x}_{1},{x}_{2},\dots ,{x}_{N}\}, {x}_{i}\in {\mathbb{R}}^{W\times H}$ as a high-dimensional representation set of the image dataset. We use the cross-entropy loss function during the training process, which reads as follows:

$$L_{cross\;entropy}=\sum\limits_{\mathrm i=1}^N\left(1_{\left\{l_i=1\right\}}\log(p(x_i))+(1-1_{\left\{l_i=1\right\}})\log(1-p(x_i))\right)$$

where ${\{l}_{i}\}$, ${l}_{i}\in${0, 1} contains the ground truth, and $p\left({x}_{i}\right)$ is the output of the deep learning model, which indicates the probability that sample ${x}_{i}$ is a schwannoma. ${1}_{\left\{{l}_{i}=1\right\}}$ is a Boolean function that reads:

$$1_{\left\{l_i=1\right\}}=\left\{\begin{array}{c}1,\;l_i=1,\;\mathrm{sample}\;x_i\;is\;schwannoma\\0,\;l_i\neq1,\;\mathrm{sample}\;x_i\;is\;ependymoma\end{array}\right.$$

When a patient needed to be diagnosed during the testing process, we used T1-weighted imaging (T1-WI), T2-weighted imaging (T2-WI), and contrast-enhanced T1-weighted imaging (CE-T1) images and input them into a dedicated classifier for the corresponding modality. For a patient with multiple image sequences, the average diagnostic probability of multiple images was used as the case-level diagnosis as shown in Fig. 2.

The diagnostic effectiveness of each classifier was evaluated in the corresponding test set, which included T1-weighted images, T2-weighted images and CE-T1 MR images. The final tumour classification model used the average probability of five models as the output and was tested on an external test set. See Fig. 2.

### Statistics and evaluation metrics

The model performance evaluation indices were recorded as true positives (TPs), false positives (FPs), true negatives (TNs) and false negatives (FNs) as follows:

$$\text{T}\text{P}={\textstyle\sum_i1_{\{l_i={\overset-l}_i\}}\times1_{\{{\overset-l}_i=1\}},\text{F}\text{P}=\textstyle\sum_i1_{\{l_i\neq{\overset-l}_i\}}\times1_{\{{\overset-l}_i=1\}},}$$

$$\mathrm{TN}={\textstyle\sum_i1_{\{l_i={\overline l}_i\}}\times1_{\{{\overline l}_i=0\}},\;\mathrm{FN}=\sum_i1_{\{l_i\neq{\overline l}_i\}}\times1_{\{{\overline l}_i=0\}}.}$$

where $${\{l}_{i}\}$$, $${l}_{i}\in$${0, 1} contains the ground truth, and $${\{\stackrel{-}{l}}_{i}\} , {\stackrel{-}{l}}_{i}\in \{0, 1\}$$ contains the predicted labels. $${1}_{\{{l}_{i}={\stackrel{-}{l}}_{i}\}}=$$$$\left\{\begin{array}{c}1 ,{l}_{i}={\stackrel{-}{l}}_{i}\\ 0 ,{l}_{i}\ne {\stackrel{-}{l}}_{i}\end{array}\right.$$ ,$${1}_{\{{l}_{i}\ne {\stackrel{-}{l}}_{i}\}}=$$$$\left\{\begin{array}{c}1 ,{l}_{i}\ne {\stackrel{-}{l}}_{i}\\ 0 ,{l}_{i}={\stackrel{-}{l}}_{i}\end{array}\right.$$ and $${1}_{\{{l}_{i}=1\}}=$$$$\left\{\begin{array}{c}1 ,{l}_{i}=1\\ 0 ,{l}_{i}\ne 1\end{array}\right.$$ are Boolean functions.

Confusion matrices were constructed for the evaluation of the deep learning model (see Fig. 6B and Supplementary Fig. 1). The predictive sensitivity and specificity of each model in the test sets were combined to evaluate its performance in forest plots. The sensitivity and specificity are defined as:

$$Sensitivity=\frac{TP}{TP+FN}=\frac{\sum_i1_{\{l_i={\overline l}_i\}}\times1_{\{{\overline l}_i=1\}}}{\sum_i1_{\{l_i=1\}}},$$

$$Specificity=\frac{TN}{TN+FP}=\frac{\sum_i1_{\{l_i={\overline l}_i\}}\times1_{\{{\overline l}_i=0\}}}{\sum_i1_{\{l_i=0\}}},$$

The diagnostic ability of the classifiers based on 3 datasets (T1-WI, T2-WI, and CE-T1) and that integrate all the modalities were compared in the SROC [29] and forest plots. All the statistical data were analysed and visualized with R (version 4.1.3) in conjunction with the meta4diag [30] and INLA R packages. In addition, Cohen’s kappa coefficients [31] for AI diagnosis and ground truth were computed.

## Results

### Implementing and testing

Our system was developed by deep learning experts at the Zhejiang University School of Mathematical Sciences. The neural network architecture was pretrained Inception-v3 with an output layer for classification tasks. The network was trained using eight 11 GB NVIDIA GeForce GTX 1080 Ti graphical processing units with a batch size of 128 and an input image size of 500 × 500 pixels using the PyTorch framework (version 1.7.1 https://www.pytorch.org) and Python (version 3.8.5 https://www.python.org). Each network was trained for 50 epochs, with an initial learning rate of 0.00005 using the Adam optimizer. The training process took approximately 7 h.

### Comparison on three CNN models

**Fig. 3:**
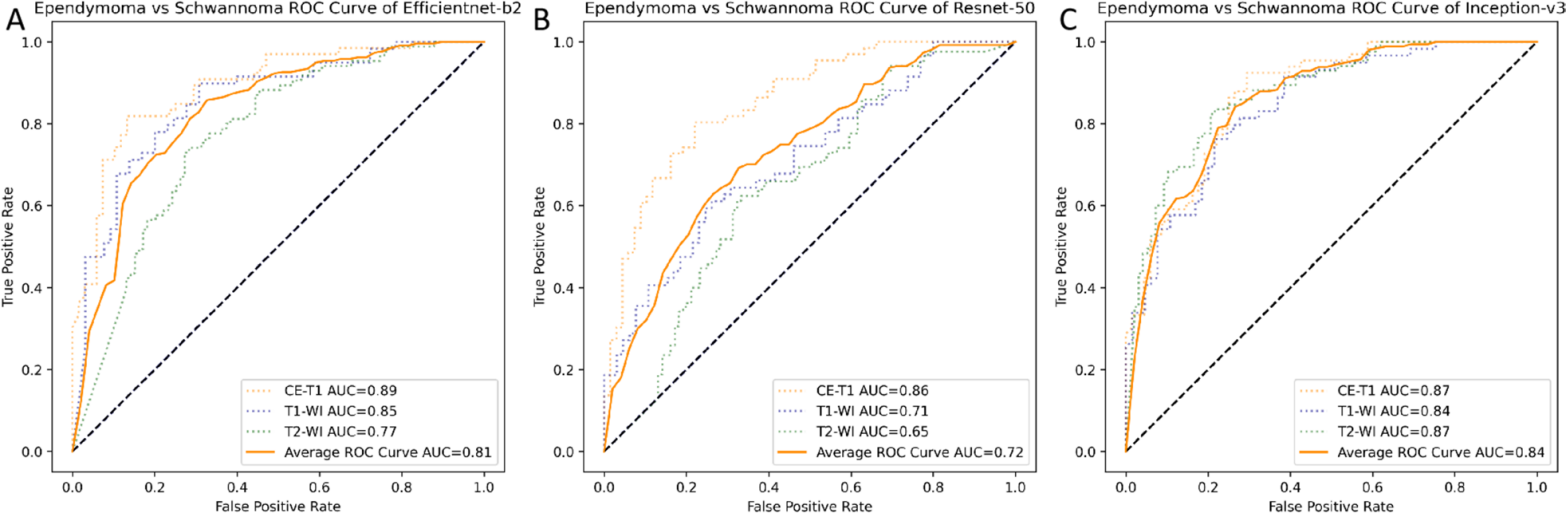
The receiver-operating curves (ROC) for the image-level external test set for 3 CNN models. The area under the curve (AUC) can summarize the diagnostic effect of different models. The CNN using **A** EfficientNet-b2 model had an average AUC of 0.81. **B** ResNet-50 model had an average AUC of 0.72. **C** Inception-v3 had an average AUC of 0.84. The blue, green and orange dot lines represent the diagnostic efficacy of T1-WI, T2-WI and CE-T1 MRI modalities, respectively

ResNet-50, EfficientNet-b2 and Inception-v3 showed their own characteristics in our study (Table 2 and Fig. 3). After several years of development, various network structures have been developed to improve the accuracy of image recognition, reduce network volume and increase efficiency. Inception-v3 [28] uses a combination of convolutional layers with different kernel sizes and pooling layers to extract features from images. EfficientNet-b2 [26] uses compound scaling, which scales the depth, width and resolution of the network to balance accuracy and efficiency. ResNet-50 [27] is a CNN architecture that introduces the concept of residual connections, which allows the network to learn residual functions rather than learning the underlying mapping directly. As shown in Fig. 3, the CNN using EfficientNet-b2 model had an average AUC of 0.81, ResNet-50 had an average AUC of 0.72 and Inception-v3 had an average AUC of 0.84. For the tasks studied in this paper, Inception-v3 generally achieved better performance.

**Table 2:** Performance evaluation of different CNN models on test images

Input	Model	Sensitivity	Specificity	Accuracy	AUC	Kappa
T1-WI	Resnet-50	55.9%	76.9%	66.9%	0.714	0.331
T2-WI	Resnet-50	40.0%	78.8%	60.9%	0.649	0.193
CE-T1	Resnet-50	74.2%	80.9%	77.6%	0.855	0.552
T1-WI	Efficientnet-b2	86.4%	69.2%	77.4%	0.854	0.552
T2-WI	Efficientnet-b2	72.9%	72.7%	72.8%	0.769	0.455
CE-T1	Efficientnet-b2	81.8%	76.5%	79.1%	0.890	0.582
T1-WI	Inception-v3	76.3%	76.9%	76.6%	0.844	0.532
T2-WI	Inception-v3	74.1%	82.5%	78.6%	0.874	0.568
CE-T1	Inception-v3	83.3%	75.0%	79.1%	0.871	0.583

### Performance of the Inception-v3 model under different MRI modalities

**Fig. 4:**
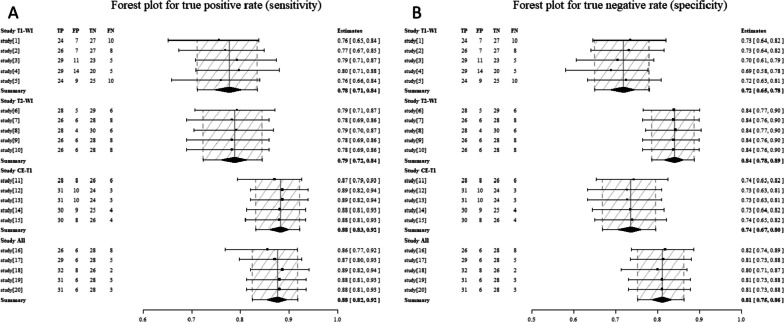
The forest plot for **A** sensitivity and **B** specificity in the external test set. Each row represents a model performance prediction, and summary represents the combined metrics for each model. The rhombus represents the combined effect value. The dashed line shadow area is the confidence interval

After selecting the optimal Inception-v3 model using images, we tested it at the case level on an external test set to analyse the performance of the AI algorithms under the different MR modalities. If the arithmetic mean of the prediction values for multiple images from a single examination was greater than 0.5, the case was marked as 1 and diagnosed as a schwannoma. Otherwise, the lesion was marked as 0 and diagnosed as ependymoma. All the evaluated indicators were analysed using the diagnostic test fourfold table. As shown in Fig. 4, in the external test dataset, the per-examination combined sensitivity was 0.78 based on T1-WI (0.71–0.84, 95%CI), 0.79 (0.72–0.84, 95%CI) based on T2-WI, 0.88 (0.83–0.92, 95%CI) based on CE-T1, and 0.88 (0.83–0.92, 95%CI) for all weighted imaging. The combined specificities based on T1-WI were 0.72 (0.66–0.78, 95% CI), 0.84 (0.78–0.89, 95% CI) based on T2-WI, 0.74 (0.67–0.80, 95% CI) for CE-T1, and 0.81 (0.76–0.86, 95% CI) for all weighted images. The better the sensitivity and specificity of a modality, the shorter the length of the confidence interval, indicating that the model’s judgements are relatively more stable. The CE-T1 modality had the highest sensitivity for ependymomas. For schwannomas, T2-weighted imaging (T2-WI) had the highest specificity. For both of them, the combined diagnostic efficiency of all the modalities was the second highest.

**Fig. 5:**
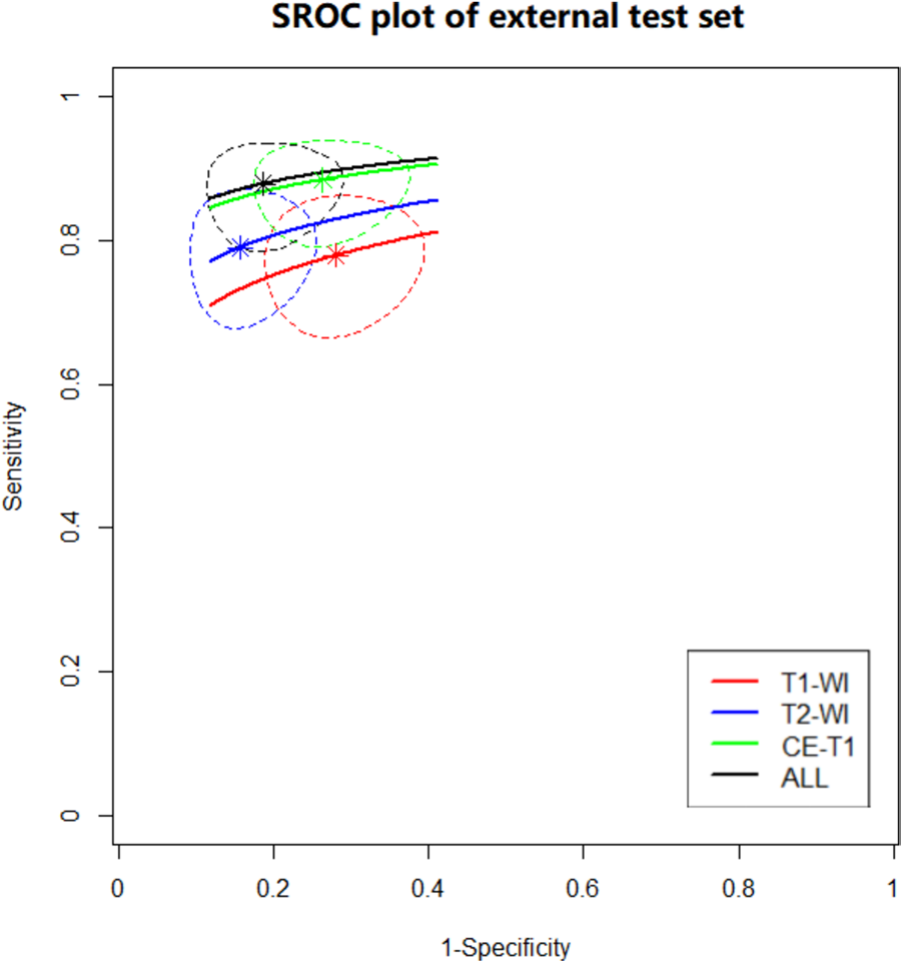
The Summary Receiver Operating Characteristic (SROC) curve of the Inception-v3 model for the test set. The horizontal axis is 1-specificity, and the vertical axis is sensitivity. The red, blue, green and black lines respectively represent the diagnostic efficacy curves of T1-WI, T2-WI, CE-T1 and All. The star points represent the combined effect value. The dashed line range is the confidence interval

The summary receiver operating characteristic (SROC) curve visually summarizes and compares the diagnostic efficiency of the four groups of diagnostic methods (T1, T2, CE-T1, and All). The SROC plot represents the relationship between sensitivity and specificity across multiple studies or datasets, providing a summary of the overall diagnostic accuracy. According to the SROC curve, a curve closer to the upper left corner indicates that the diagnostic performance of the model is better. As shown in Fig. 5, the best diagnostic method was achieved by the group based on all the images. The second was CE-T1 images. The performance of the different MRI modalities for Inception-v3 is shown in Table 3.

**Table 3:** Performance evaluation of different MRI modalities on Inception-v3

Input	Sensitivity	Specificity	Accuracy	AUC	Kappa
T1-WI	73.5%	73.5%	73.5%	0.813	0.471
T2-WI	76.5%	85.3%	80.9%	0.912	0.618
CE-T1	88.2%	76.5%	82.4%	0.888	0.647
ALL	94.1%	79.4%	86.8%	0.928	0.735

In summary, the combination of multiple MRI modalities yielded better diagnostic efficiency than a single modality.

#### CNN can achieve differential diagnosis based on image feature regions

**Fig. 6:**
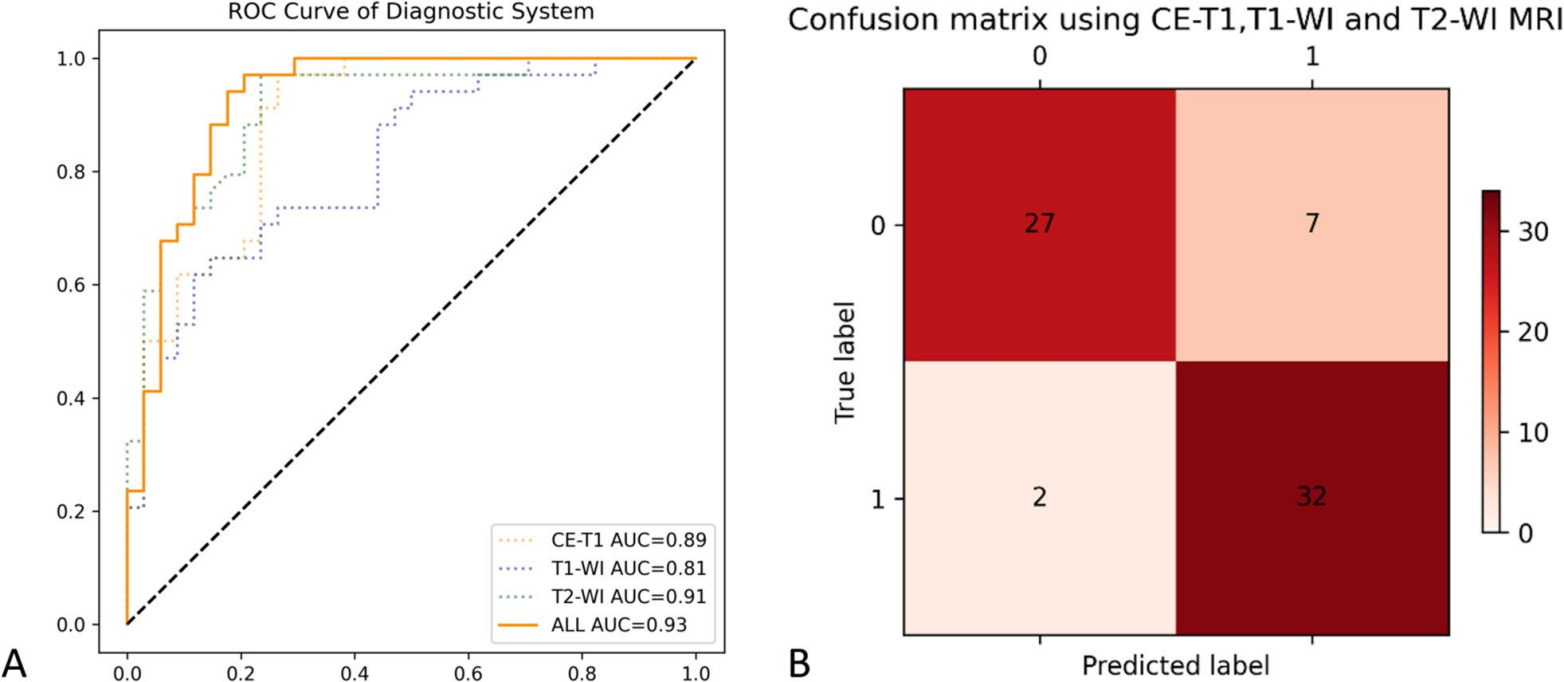
ROC curve and confusion matrix of the diagnostic system. **A** The blue, green and orange dot lines represent the diagnostic efficacy of T1-WI, T2-WI and CE-T1 MRI modalities, respectively. **B** The horizontal axis represents the predicted values, and the vertical axis represents the true values. The four quadrants (from top to bottom, left to right) represent true negative, false negative, false positive, and true positive, respectively. The shades of red indicate the frequency of occurrences

As shown by the ROC curve and confusion matrix (Fig. 6), our diagnostic system achieved an AUC of 0.93 and an accuracy of 0.87, indicating promisingly high diagnostic performance.

**Fig. 7:**
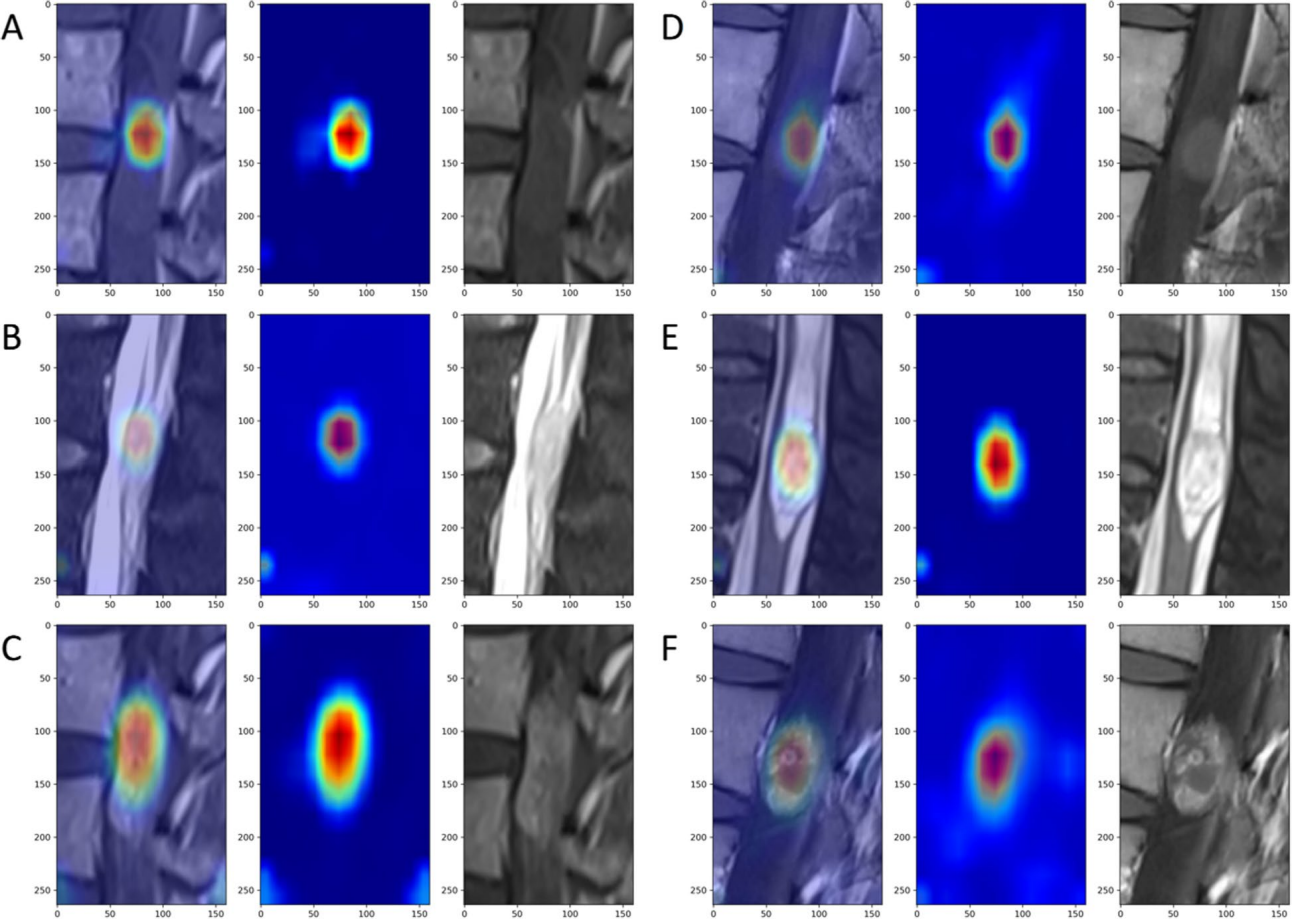
Regions of interest of the diagnostic system computed by Grad-CAM. **A**, **B** and **C** are ependymomas, corresponding to T1-WI, T2-WI, and CE-T1, respectively. **D**, **E** and **F** are schwannomas, corresponding to T1-WI, T2-WI, and CE-T1, respectively. The areas that appear more red indicate that the model pays more attention to those regions

We used Grad-CAM (gradient-weighted class activation mapping) [32] to visualize which regions in the neural network contributed more to the classification results. We present our results in the form of heatmaps in Fig. 7. In the images, the areas that appear redder indicate that the model pays more attention to those regions. Importantly, we did not use supervised learning to perform tumour segmentation. Grad-CAM generates class activation maps (CAMs) by utilizing the feature maps of deep convolutional neural networks, helping us understand how neural networks make decisions in image classification tasks. We can see that the model pays more attention to the solid parts of the tumour.

### Data availability

We will make the model parameters of this study publicly available on GitHub at https://github.com/SLYXDWL/SpineTumorClassification.git.

## Discussion

Successfully identifying FTEs from schwannomas using preoperative imaging can be beneficial in clinical practice. Considering the necessity of en bloc resection for ependymomas, surgeons tend to increase the window area to create a wider surgical field. However, schwannomas can be resected using minimum invasive access after debulking, protecting weight-bearing spine structures and creating smaller skin incisions. In this case, our diagnostic system can be very helpful for determining the pathological type of tumour in advance when choosing the procedure.

The FTEs in our definition included spinal ependymoma (SPE) and myxopapillary ependymoma (MPE) without MYCN amplification according to the 2021 CNS WHO classification [24]. Compared with the 2016 CNS WHO classification, MPE is now regarded as CNS WHO grade 2 rather than 1 because the likelihood of recurrence and mean interval of recurrence [7, 33–37] are now considered similar to those of SPE (WHO II). We found compelling evidence that en bloc GTR significantly reduces the recurrence of FTEs, regardless of the SPE or MPE subtype, based on a 20-year case study [10]. Although large-scale prospective studies have not been conducted, there is substantial retrospective literature evidence indicating that GTR via an en bloc technique significantly decreases recurrence and prevents repeated surgeries [38–40]. Our diagnostic system’s ability to identify ependymomas prior to surgery can assist doctors in formulating surgical plans for en bloc resection. Before or during the dissection of FTEs, conus medullaris and cauda equina injuries can occur, leading to a complex syndrome of motor, sensory, and autonomic impairments [41]. Symptoms such as sexual dysfunction and urinary or bowel incontinence drastically affect patients’ health and quality of life and impact patient satisfaction with surgery. Accurate preoperative diagnosis can provide patients and their families with reasonable expectations of surgical outcomes and inform patients of potential complications. Our diagnostic system can be very important for the shared decision-making of patients and clinicians about treatment strategies.

In this study, we confirmed the possibility of differentiating FTEs from schwannomas via a CNN. With an AUC of 0.93 and an accuracy of 0.87, as demonstrated by the ROC curve and confusion matrix (Fig. 6), our diagnostic system exhibited a high diagnostic performance. We showed that the results of our model can provide objective and reproducible second opinions to assist radiologists and surgeons in making correct decisions. To our knowledge, this is the very first study to construct a CNN diagnostic model utilizing MR images to distinguish between FTEs and schwannomas.

We established a relatively large training set based on the strengths of our neurosurgery centre. The sex distribution of patients matched that in previous literature [2, 24]. We also employed 5-fold cross-validation to validate the developed models. A good model requires good generalization capacity, which means that it must perform well on both training data and new datasets. Fivefold cross-validation reduces variance by averaging the results of 5 different training groups. Therefore, the performance of the model is no longer sensitive to the division of data compared to that of holdout cross-validation. The 10-patient held-out test set was still from our institution but was not included in the training or validation phase; moreover, the data were randomly acquired from 9 MRI machines to avoid overfitting.

Interpretation of machine learning methods with complex internal structures has received a growing amount of scholarly attention. We conducted preliminary experiments using Grad-CAM to visualize our model’s attention to different regions of the images. When the model made correct predictions, we observed that its attention was often focused on the tumour mass. Like human observers, CNN models tend to rely on features within the tumour mass for classification. The features of the results of the present study can inspire us to explain the radiological features of these two tumours. As shown in Fig. 4, the enhanced sequence was most sensitive for identifying schwannomas. The T2-WI sequence is most sensitive to FTEs. These characteristics may be related to the vascular richness of the two kinds of tumours [42, 43]. Kenyu et al. noted that MRI signal patterns in T2-weighted (T2-W) hyperintense areas are based on cell density (mucin or free water content) and mesh patterns in the interstitial tumour space [44]. After all, the exact features learned by the deep learning model could not be revealed due to its “black-box” nature and require further study.

We also observed that the region of interest in the tumour area in the model was generally smaller than the actual extent of the tumour across multiple magnetic resonance imaging modalities. In some cases, the model also focused on intervertebral discs, possibly due to their similarity in magnetic resonance signals to tumours. These findings suggest that relying solely on pathological labels has limitations in accurately determining tumour boundaries. Our model lacks detection and segmentation modules, which limits the use of a comprehensive intelligent diagnostic system.

Limitations in terms of patient numbers, which is a drawback of this research, are inherent to the tumour types and therefore unavoidable to at least a degree. We employed figure processing and 5-fold cross-validation due to the limited amount of data for this task. However, an international, multicentre, larger sample study is the direction of our future efforts. On the premise of a larger dataset, MPE and SPE can also be more clearly distinguished. There was also a difference in CT density between the two tumours. The integration of CT sequences into a deep learning model may achieve improved diagnostic performance. In the future, it will be possible to distinguish between more lesions throughout different spinal cord segments, such as meningiomas, astrocytomas, epidermoid cysts, haemangioblastomas, metastatic tumours and syringomyelia.

This study used single-layer 2-dimensional sagittal images. In the real clinical process, physicians read all the levels and orientations of images to make a comprehensive judgement. In the machine learning process, the characteristic connections between different layers of the tumour are missing. The algorithm for 3D image classification may further improve diagnostic efficiency.

## Conclusion

We present a deep learning model for the classification of filum terminale ependymomas and schwannomas with the potential to augment clinical diagnosis. Our work represents the application of artificial intelligence in medicine and encourages future research in this area.

### Supplementary Information

**Supplementary Material 1.**

## Data Availability

The datasets generated during and/or analysed during the current study are available from the corresponding author on reasonable request.
