# Supplementary material for: Convolutional neural network-based magnetic resonance image differentiation of filum terminale ependymomas from schwannomas
Source: BMC Cancer. 2024 Mar 19;24:350. doi: 10.1186/s12885-024-12023-0 (PMC10949807; doi:10.1186/s12885-024-12023-0)
Supplement: Supplementary file 1 — Supplementary Material 1. [file 12885_2024_12023_MOESM1_ESM.docx]

**Supplementary material**


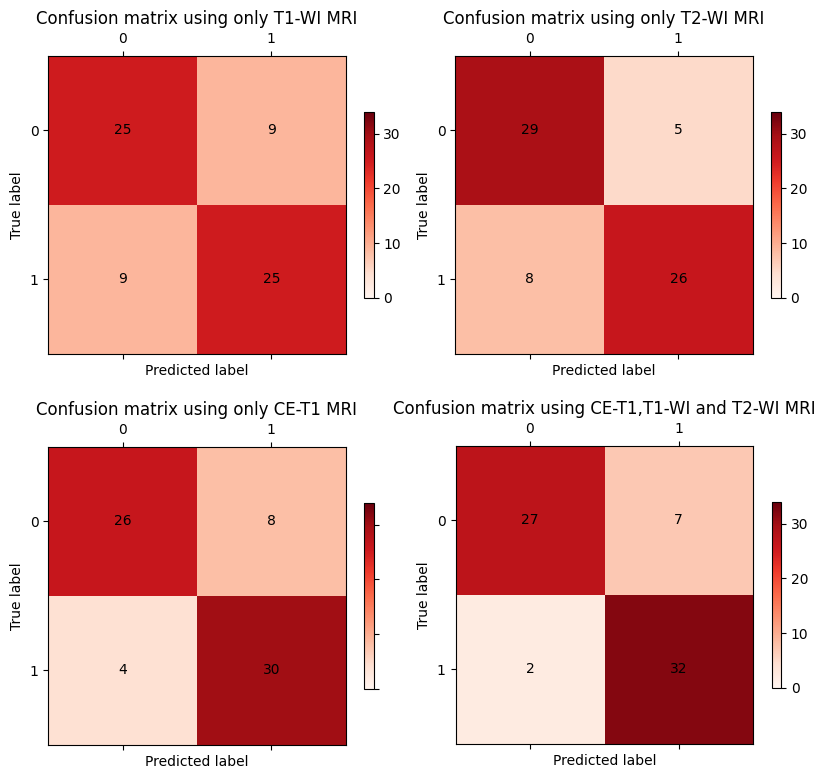


Supplement figure 1. The confusion matrix for the case-level external test set. If the arithmetic mean of prediction values for multiple images of one examination is greater than 0.5, the case would be labelled as 1 and diagnosed as schwannoma. Otherwise, it would be marked as 0 and diagnosed as ependymoma.


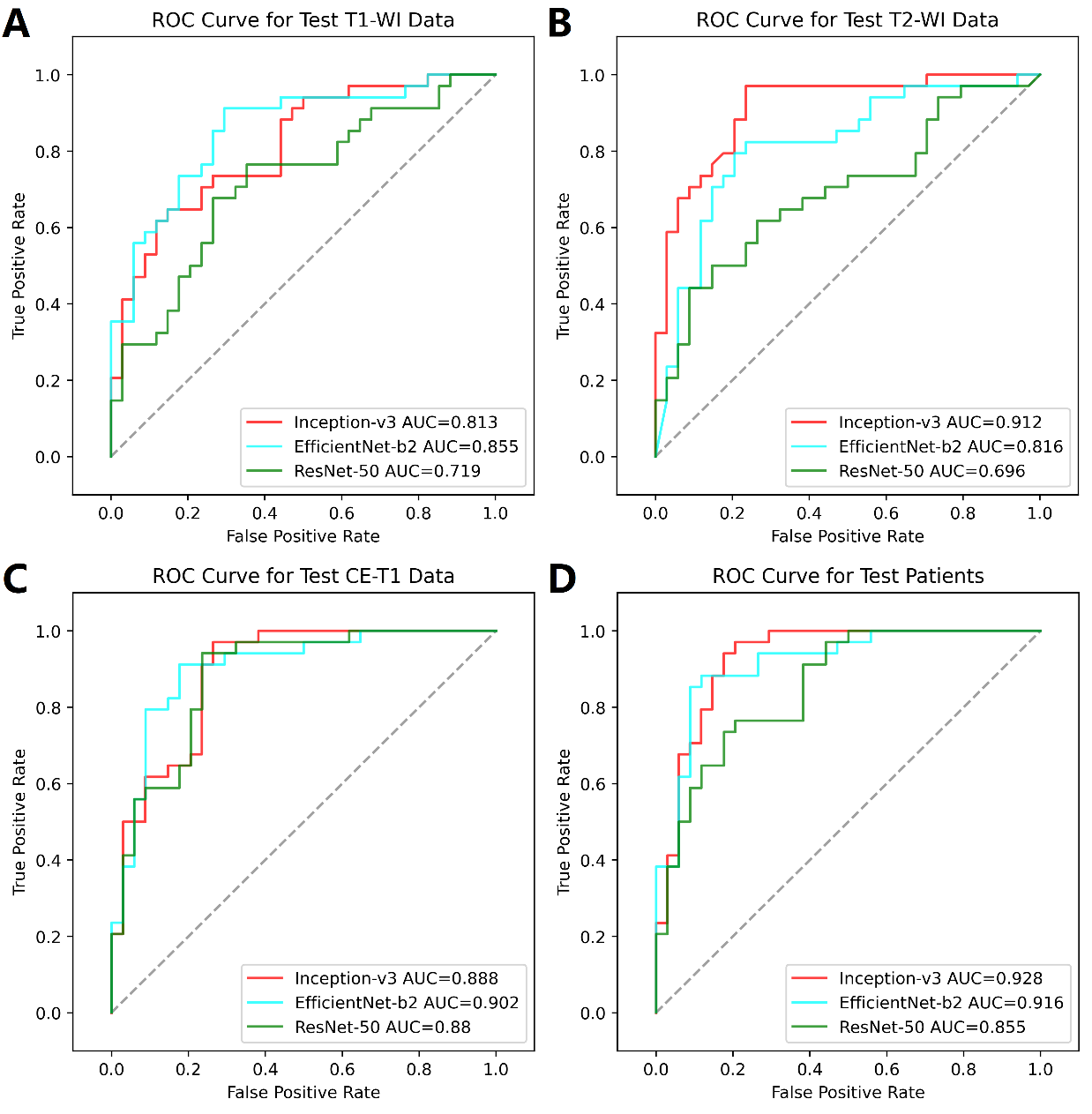


Supplement figure 2. The receiver-operating curves (ROC) of 3 types of CNN models for the patient-level external test set. The area under the curve (AUC) can summarize the diagnostic effect of different models. The red, blue, green lines represent the diagnostic efficacy of Inception-v3, EfficientNet-b2 and ResNet-50, respectively.

| **Input** | **Model** | **Sensitivity** | **Specificity** | **Accuracy** | **AUC** | **Kappa** |
| --- | --- | --- | --- | --- | --- | --- |
| T1-WI | Resnet-50 | 61.8% | 73.5% | 67.6% | 0.719 | 0.353 |
| T2-WI | Resnet-50 | 44.1% | 88.2% | 66.2% | 0.696 | 0.324 |
| CE-T1 | Resnet-50 | 82.4% | 76.5% | 79.4% | 0.880 | 0.588 |
| ALL | Resnet-50 | 58.8% | 88.2% | 73.5% | 0.855 | 0.471 |
| T1-WI | Efficientnet-b2 | 88.2% | 70.6% | 79.4% | 0.855 | 0.588 |
| T2-WI | Efficientnet-b2 | 79.4% | 79.4% | 79.4% | 0.816 | 0.588 |
| CE-T1 | Efficientnet-b2 | 82.4% | 82.4% | 82.4% | 0.902 | 0.647 |
| ALL | Efficientnet-b2 | 88.2% | 79.4% | 83.8% | 0.916 | 0.676 |
| T1-WI | Inception-v3 | 73.5% | 73.5% | 73.5% | 0.813 | 0.471 |
| T2-WI | Inception-v3 | 76.5% | 85.3% | 80.9% | 0.912 | 0.618 |
| CE-T1 | Inception-v3 | 88.2% | 76.5% | 82.4% | 0.888 | 0.647 |
| ALL | Inception-v3 | 94.1% | 79.4% | 86.8% | 0.928 | 0.735 |

Supplement table 1. Performance evaluation of different CNN models on test patients. We use inception-v3 to build our diagnostic system, so only the diagnostic efficacy of inception-v3 in cases is shown in the manuscript. In fact, we also built diagnostic systems using different neural networks and tested the effectiveness of case level diagnosis. The optimal model Inception-v3 selected from the validation image also showed the best performance in case level diagnostic tasks.
